# Improved A* Path Planning Method Based on the Grid Map

**DOI:** 10.3390/s22166198

**Published:** 2022-08-18

**Authors:** Yangqi Ou, Yuexin Fan, Xinglan Zhang, Yanhua Lin, Weijing Yang

**Affiliations:** 1 College of Automation, Chongqing University, Chongqing 400044, China; 2 College of Computer Science and Engineering, Chongqing University of Technology, Chongqing 400054, China

**Keywords:** mobile robots, improved A* algorithm, path searching, Hdl_graph_slam mapping

## Abstract

In obstacle spatial path planning, the traditional A* algorithm has the problem of too many turning points and slow search speed. With this in mind, a path planning method that improves the A* (A-Star) algorithm is proposed. The mobile robot platform was equipped with a lidar and inertial measurement unit (IMU). The Hdl_graph_slam mapping algorithm was used to construct a two-dimensional grid map, and the improved A* algorithm was used for path planning of the mobile robot. The algorithm introduced the path smoothing strategy and safety protection mechanism, and it eliminated redundant points and minimal corner points by judging whether there were obstacles in the connection of two path nodes. The algorithm effectively improved the smoothness of the path and facilitated the robot to move in the actual operation. It could avoid the wear of the robot by expanding obstacles and improving the safety performance of the robot. Subsequently, the algorithm introduced the steering cost model and the adaptive cost function to improve the search efficiency, making the search purposeful and effective. Lastly, the effectiveness of the proposed algorithm was verified by experiments. The average path search time was reduced by 13%. The average search extension node was reduced by 11%. The problems of too many turning points and slow search speed of traditional A* algorithm in path planning were improved.

## 1. Introduction

When a robot performs a task, planning the optimal or suboptimal movement path from the starting point to the target point in the obstacle space is a core problem. For traditional path planning algorithms, when planning paths for obstacle spaces, there are too many turning points and slow search speeds [1]. The map environment model is needed to study the path planning problem; the mobile robot is equipped with multiple sensors to collect environmental information, and the map model is carried out by simultaneous positioning and mapping (SLAM). The most commonly used methods for representing map models are the grid method, topology method, and viewable method. Common path programming algorithms include the A* algorithm, Dijkstra algorithm, D*Lite algorithm, genetic algorithm, and artificial potential field [2,3].

For different path planning algorithms, the Dijkstra algorithm needs to search for many unnecessary inflection points in the execution process, which wastes computer resources [4,5]. Due to the error of the heuristic value of the D* Lite algorithm, the planned path crosses obstacles, which is unfavorable for actual mobile robot operation [6,7]. The path search of the genetic algorithm has a slow convergence speed and poor local search ability [8,9]. When the target point is near the obstacle, the artificial potential field method is prone to falling into local oscillation, becoming unable find the final path [10]. The A* algorithm establishes the cost function between the starting point and the target point through the heuristic function to make the search purposeful and accelerate the search speed [11,12].

Researchers have performed extensive research to improve path planning. The authors of [13] proposed the Gmapping algorithm based on the particle filter to construct an environmental grid map, and they adopted the A* algorithm to realize global path planning of the mobile robot. However, the problems of excessive path turning points and slow search speed were not considered; hence, the method was not applicable. The authors of [14] proposed an improved cartographer algorithm based on graph optimization to create grid maps, and the cost functions of predictive distance and dynamic measurement heuristic were introduced into the A* algorithm. This approach effectively solved the problem of the A* algorithm easily falling into a local optimum due to many extended grids in path planning. However, the cartographer algorithm is time-consuming compared with the Hdl_graph_slam algorithm. The authors of [15] presented an improved ant colony algorithm, by changing the pheromone update methods and effectively reducing initial path planning of the blind search to improve the search efficiency. The authors of [16] put forward an improved A* algorithm, introducing the extension distance, bidirectional search, and smooth path; the proposed method improved the efficiency of path planning, as well as reduced the search grid number and right-angle turn number.

Inspired by the above discussion, this paper proposes an improved A* algorithm and proposes some new methods to further improve the performance. The goal of this paper was to achieve the global path planning of mobile robots, focusing on accelerating the search speed and improving the overall performance of the algorithm. The main contributions of this paper are as follows:

(1) The path smoothing strategy is introduced to eliminate redundant points and inflection points and reduce the frequent direction changes of mobile robots during walking;
(2) The safety protection mechanism is increased to avoid damage caused by friction between the mobile robot and obstacles in the process of walking;
(3) The steering cost model and adaptive cost function are added to reduce the search time and improve the efficiency of the algorithm;
(4) Autonomous path planning of mobile robots is realized by combining the mapping algorithm of Hdl_graph_slam with the improved A* path planning algorithm.
(5) Lastly, the comparative experimental results are given to prove the superiority of the algorithm.

The remainder of the paper is organized as follows: the Hdl_graph_slam environment modeling is described in Section 2, the improved path planning method is introduced in Section 3, the comparative experiment-level results are introduced and discussed in Section 4, and the conclusions are presented in Section 5.

## 2. Hdl_Graph_Slam Environment Modeling

Hdl_graph_slam is a graph-building method based on graph optimization. The mobile robot platform is equipped with lidar and IMU sensors to collect environmental information by subscribing to sensor topics. It uses IMU data to correct the distortion of point cloud data obtained by lidar and then performs ConditionalRemoval, VoxelGrid, and RadiusOutlierRemoval to remove noise points and outliers. The processed point cloud information is passed to the point cloud matching module and floor detection module. The point cloud matching module adopts the multi-thread algorithm of normal distribution transform (NDT_OMP) to match the relative pose of two adjacent point clouds. The NDT_OMP algorithm describes the local characteristics of the point clouds as a probability density function, which reduces the memory cost of the storing point cloud information as a function of the coordinate value. The floor detection module adds a ground constraint to the mapping module to effectively reduce the elevation error [17]. The point cloud data processed using the point cloud matching module and floor detection module are input into the map optimization module to complete the map construction, and the grid map is constructed using octree.

### 2.1. Point Cloud Matching

**Figure 1:**
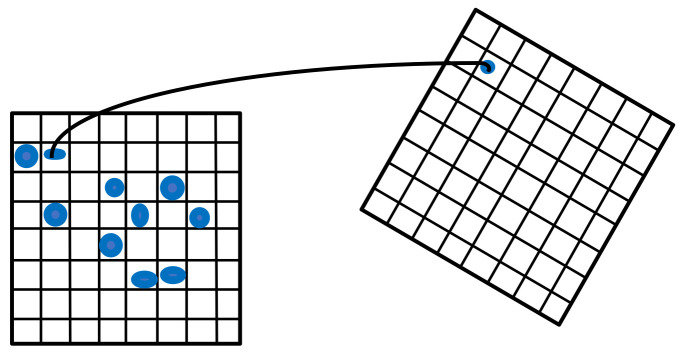
NDT diagram. The blue dots show the various normal distributions.

As shown in Figure 1, a normal distribution indicates that the point cloud data can describe the local morphology of the point cloud.

By calculating the mean and covariance in each grid in the reference point cloud on the left side of Figure 1,

(1) $$q=\frac{1}{m}\sum_{i}X_{i},$$

(2) $$\sum =\frac{1}{m}\sum_{i}\left(X_{i}-q\right){\left(X_{i}-q\right)}^{T},$$

where $X_{i}$ represents the coordinates of the point cloud, and the blue ellipse is the ellipse fitted with a normal distribution. In Figure 1, the right side represents the target point cloud scanned at the current moment, calculates the coordinates of the target point cloud in the reference point cloud, and determines the corresponding normal distribution of the target point cloud in the reference point cloud. Coordinates of the target point cloud with respect to the reference point cloud are expressed as

(3) $$X_{i}^{\prime }=T(X_{i},P),$$

(4) $$P={\left(p_{i}\right)}_{i=1...3}^{i}={\left(t_{x},t_{y},\phi \right)}^{T},$$

where T represents the process of converting the target point cloud coordinates to the reference point cloud coordinates, P represents the transformation matrix of the target point cloud to the reference point cloud, and the objective function is expressed as

(5) $$score(P)=\sum_{i}\exp(\frac{-{\left(X_{i}^{\prime }-q_{i}\right)}^{T}\sum_{i}^{-1}\left(X_{i}^{\prime }-q_{i}\right)}{2}).$$

Equation (5) can be abbreviated as

(6) $$s=-\exp\frac{-q^{t}\sum_{}^{-1}q}{2},$$

(7) $$q=X_{i}^{\prime }-q_{i}.$$

The optimization problem is usually described as a minimization problem. The Newton algorithm is used to iteratively identify the parameters that minimize the function, and the optimal parameters are calculated by iteratively solving the following equation:

(8) HΔP=−g,

where g is the transpose gradient of score(P), and H is the Hessian matrix of score(P) [18].

### 2.2. Floor Detection

In a flat environment, the mobile robot moves without much jitter, and the elevation error can be reduced by adding floor detection constraints. Due to a large number of point clouds, all point clouds do not need to participate in the calculation of the floor detection algorithm. To save computing resources, the floor is detected every 10 s, and the floor detection algorithm firstly performs point cloud segmentation on the filtered point cloud, which is segmented twice in total to extract the point cloud data in the specified range above and below the sensor [19,20]. Floor detection assumes that there is global consistent ground, and the RANSAC algorithm is used to detect floor features and correct pose estimation.

**Figure 2:**
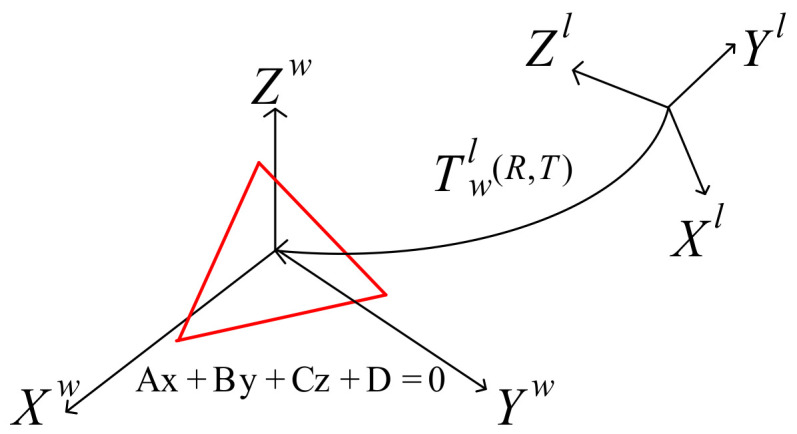
Conversion of world coordinate system to lidar coordinate system. A red triangle is a plane in three dimensions.

As shown in Figure 2, it is assumed that, in the world coordinate system $X^{w}Y^{w}Z^{w}$, the general equation of the global plane π1 is

(9) Ax+By+Cz+D=0,

the lidar coordinate system is $X^{l}Y^{l}Z^{l}$, and the transformation from the world coordinate system to lidar is $T_{w}^{l}(R,T)$.

According to the general equation of the global plane and the transformation matrix from the world coordinate system to the lidar coordinate system, the parameter equation of the global plane in the lidar coordinate system can be solved. Assuming the normal vector $\vec{n}=(x_{n},y_{n},z_{n})$ of the plane at a point $a(x_{a},y_{a},z_{a})$ on the global ground, the parametric equation of the global plane can be obtained as

(10) $$x_{n}x+y_{n}y+z_{n}z-(x_{n}x_{a}+y_{n}y_{a}+z_{n}z_{a})=0.$$

The plane normal vector $\vec{n}$ in radar coordinate system can be expressed as

(11) $$\vec{n^{\prime }}=R{\left(x_{n},y_{n},z_{n}\right)}^{T}.$$

Point $a(x_{a},y_{a},z_{a})$ is expressed in the lidar coordinate system as

(12) $$a^{\prime }(x_{a}^{\prime },y_{a}^{\prime }{,z}_{a}^{\prime })=Ra(x_{a},y_{a},z_{a})+T.$$

The expression of the global ground in the lidar coordinate system is

(13) $$x_{n}^{\prime }x+y_{n}^{\prime }y+z_{n}^{\prime }z-(x_{n}^{\prime }x_{a}^{\prime }+y_{n}^{\prime }y_{a}^{\prime }+z_{n}^{\prime }z_{a}^{\prime })=0.$$

The floor detection equation fitted by the RANSAC function is as follows:

(14) $$x_{d}x+y_{d}x+z_{d}x+D_{d}=0.$$

**Figure 3:**
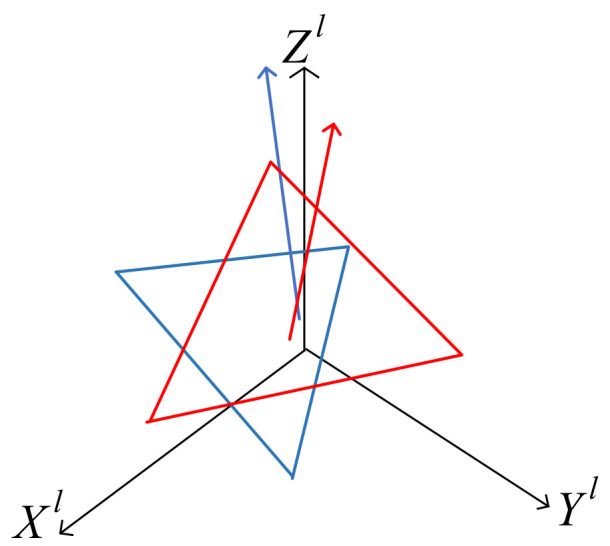
Two fitting planes in the lidar coordinate system.

In the lidar coordinate system, two plane equations are obtained. Due to measurement error, the coefficients of the two plane equations are different; hence, it is necessary to define an error equation to measure the degree of inconsistency. In Figure 3, there are two fitted planes in the lidar coordinate system. The blue plane represents the plane of global uniform ground conversion to the radar coordinate system, and the red plane represents the plane of RANSAC fitting. Their normal vectors correspond to vectors of the same color [21].

To express this error quantitatively, a rotation matrix is constructed. The plane normal vector of the globally uniform ground in the lidar coordinate system is rotated to the *X*-axis as follows:

(15) $$\left[\begin{array}{l} 1\\ 0\\ 0 \end{array}\right]=R_{x}\left[\begin{array}{l} x_{n}^{\prime }\\ y_{n}^{\prime }\\ z_{n}^{\prime } \end{array}\right].$$

This rotation angle is denoted as α, and the rotation matrix is applied to the plane equation fitted by RANSAC algorithm as follows:

(16) $$\left[\begin{array}{l} x_{d}^{\prime }\\ y_{d}^{\prime }\\ z_{d}^{\prime } \end{array}\right]=R_{x}\left[\begin{array}{l} x_{d}\\ y_{d}\\ z_{d} \end{array}\right].$$

This rotation angle is denoted as β, and the errors represented by the included angles of the two transformed normal vectors, α and β, are expressed as

(17) $$\alpha =\arctan2(y_{d}^{\prime },x_{d}^{\prime }),$$

(18) $$\beta =\arctan2(z_{d}^{\prime },\sqrt{{y_{d}^{\prime }}^{2}+{x_{d}^{\prime }}^{2}}).$$

If the two planes are parallel, they are represented by the intercept between the two planes, denoting $D^{\prime }$ as the intercept of the global ground fitting plane in the lidar coordinate system; then, the error function of the system can be expressed as

(19) $$D^{\prime }=-(x_{n}^{\prime }x_{a}^{\prime }+y_{n}^{\prime }y_{a}^{\prime }+z_{n}^{\prime }z_{a}^{\prime }),$$

(20) $$e_{rr}={\left[\alpha ,\beta ,D^{\prime },-D_{d}\right]}^{T},$$

(21) $$e={e_{rr}}^{T}\Omega e_{rr}.$$

### 2.3. Using Octree to Build Grid Maps

**Figure 4:**
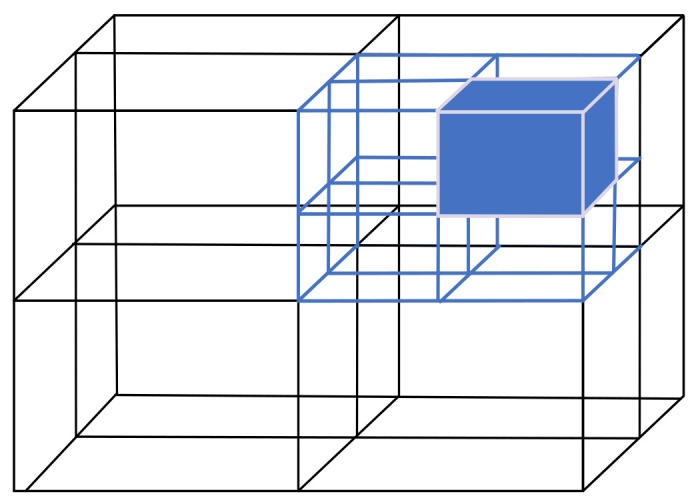
Schematic diagram of octree.

In this experiment, an octree was used to construct a map model, which can not only compress the size of the point cloud data but also quickly search the state of the grid. The octree divides the whole point cloud map into eight equal parts. The schematic diagram of the octree is shown in Figure 4.

The whole point cloud map is taken as the root node, and each root node is extended to eight subnodes, where each node represents a small cube recursively to the smallest sub-node in turn. The state of the grid is represented by the probability that each node is occupied, and the search for a node is stopped when the probability of all the children of the node is equal. In order to improve the real-time performance of the system, key frames were selected in this experiment to construct an octree map [22,23].

## 3. Path Planning for Mobile Robots

The A* algorithm makes the search purposeful by establishing the cost function between the starting point and the target point. The traditional A* algorithm divides the cost function into two sections, i.e., the cost from the starting point to the current grid and the cost from the current grid to the target point. It is a heuristic algorithm to select the grid with the lowest generation value as the extended grid by calculating the total cost function. Compared with the Dijkstra algorithm, the A* algorithm has an advantage in search speed in terms of time, but there are too many turning points, leading to an unsmooth planned path. Too many turning points in the actual operation of mobile robots will reduce the efficiency of operation and increase the wear of machine parts. At the same time, because the A* algorithm seeks the optimal or suboptimal path from the target point, the path planned by the A* algorithm always fits obstacles, resulting in the mobile robot encountering obstacles in the process of walking. Therefore, the A* algorithm needs to be improved.

A safety protection mechanism is introduced to expand the two-dimensional grid map, where the expansion distance is equal to the diameter of the robot. The path planned by the restricted path planning algorithm does not fit the edge of obstacles to protect the mobile robot from wear.

**Figure 5:**
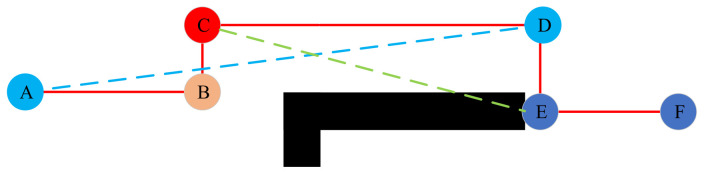
Elimination of redundant points. The letters A–F represent nodes in the planned path, red lines represent planned paths, blue and green lines represent lines between nodes, and black represents obstacles.

The path smoothing strategy is introduced to determine whether there is obstacle information on the line between the planned path nodes. If there is no obstacle on the line between two points, it indicates that the path node between two points is redundant and can be removed. If there are obstacles to the connection between two points, the intermediate node cannot be removed, as shown in Figure 5.

The solid red line in Figure 5 represents the path. The connection between nodes C and E crosses an obstacle; hence, node D cannot be deleted. The line between nodes A and D does not cross obstacles; thus, nodes B and C between nodes A and D are redundant nodes. They can be deleted from the path node set to increase the smoothness of the path and shorten the total path distance.

**Figure 6:**
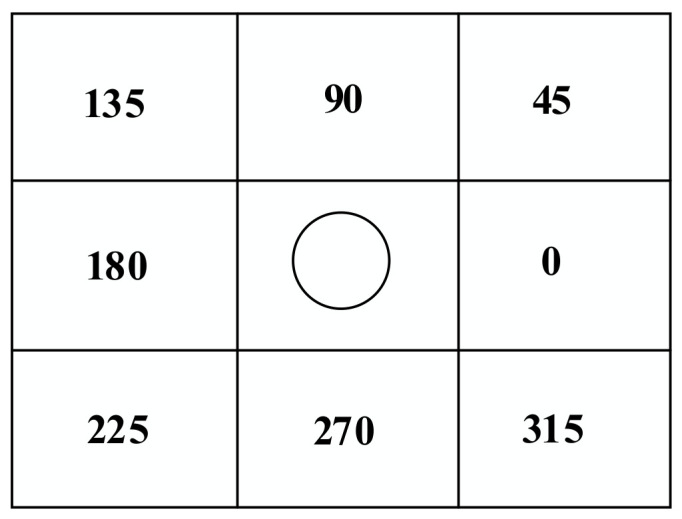
Angle of steering cost model of expansion node.

**Figure 7:**
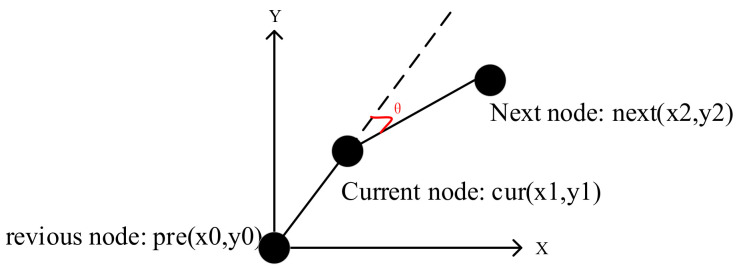
Calculation model of steering cost model angle.

When the mobile robot is in actual operation, the turning time of the inflection point cannot be ignored. By adding the steering cost model into the total cost function, the planned path is in line with the operability of the actual mobile robot. The mobile robot is set to turn toward the center point of the grid each time, and the steering cost angle model is as shown in Figure 6 and Figure 7.

The relationship between coordinate transformation and steering angle is shown in Table 1, where the *X*-axis change refers to the changes in the *X*-axis coordinates of the robot at different moments, and the *Y*-axis change refers to the changes in the *Y*-axis coordinates of the robot at different moments:

The coordinate transformation of nodes at the current moment and the previous moment is denoted as follows:

(22) $$\Delta x=x_{1}-x_{0},$$

(23) $$\Delta y=y_{1}-y_{0}.$$

The current moment and the previous moment are denoted as α according to the steering angle corresponding to Table 1.

The coordinate transformation of nodes at the next moment and the current moment is denoted as follows:

(24) $$\Delta x=x_{2}-x_{1},$$

(25) $$\Delta y=y_{2}-y_{1}.$$

The next moment and the current moment are denoted as β according to the steering angle corresponding to Table 1.

The formula of the steering cost model is as follows:

(26) $$turn\text{\_}w=\left\{\begin{array}{l} k\frac{\alpha -\beta }{45},\alpha -\beta \leq 180{}^{\circ}\\ k\frac{\alpha -\beta -180}{45},\alpha -\beta >180{}^{\circ} \end{array}\right.,$$

where *k* represents the proportionality coefficient, and *k* = 10 in this experiment. The h(n) of the total cost function uses the Manhattan distance to estimate the cost of the current node from the target node; this, h(n) has a great influence on the performance of the path search algorithm. The search performance of the algorithm can be improved by increasing the weight of h(n). In cases where g(n) and turn_w(n) are defined, the value of h(n) plays an important role. When the extended node is closer to the target node, the corresponding value of h(n) is smaller. A dynamic balance method is adopted to determine the weight of h(n) and increase the efficiency of the algorithm. When the node is far from the target point, the weight is large, and, when the node is close to the target point, the weight is small. The adaptive weight adjustment of h(n) is realized, and the search efficiency of the robot in different positions is improved. The improved expression of the total cost function in this paper is as follows:

(27) $$f(n)=g(n)+turn\text{\_}w(n)+(1+\frac{d}{D})\times h(n),$$

where d represents the Euclidean distance from the current node to the target node, and D represents the Euclidean distance from the start node to the target node.

## 4. Experiment

**Figure 8:**
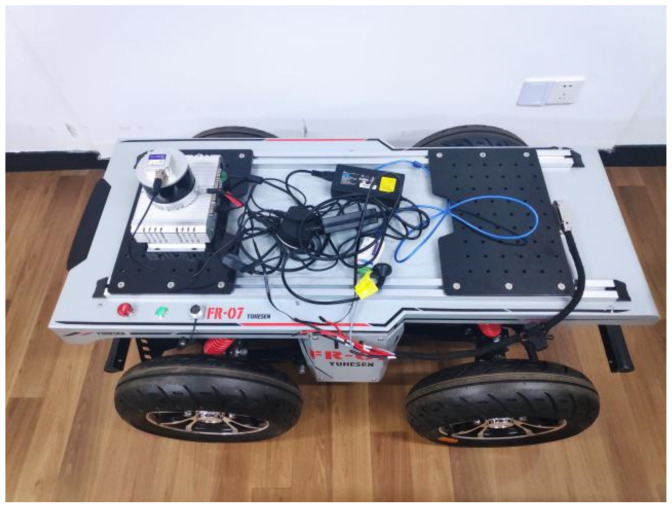
Experimental device.

The emulation configuration used in this work was as follows: Ubuntu18.04; Ros-melodic; Quadruped driven mobile robot; Hesai PandarXT-16; Lpms-IG1-4 85 IMU. The horizontal angular resolution of the lidar sensor was 0.09°, approximately 60,000 point cloud data were collected per frame, and the angular resolution of the IMU was 0.01°. The experimental device of this experiment is shown in Figure 8. Through data collection in the indoor environment, the total data size was 2.8 GB, the total duration was 93.19 s, and the total distance was 20.36 m.

### 4.1. Environmental Modeling

**Figure 9:**
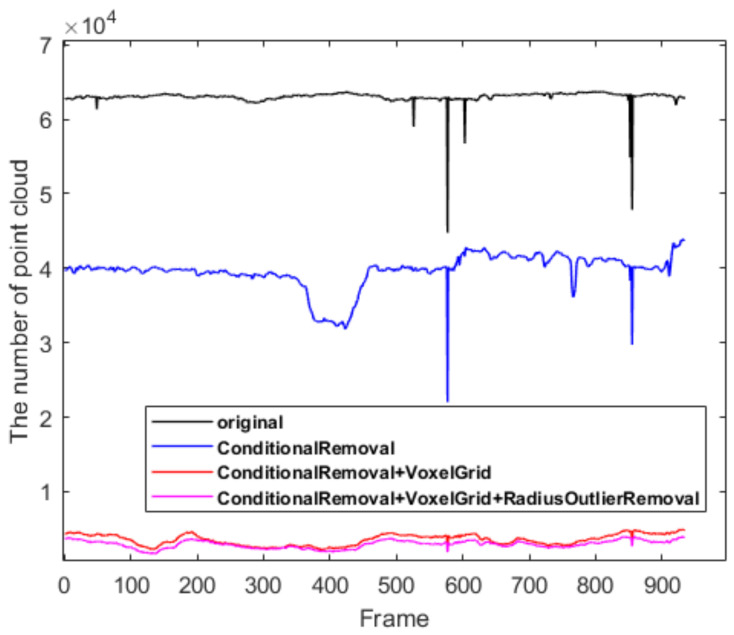
Rendering of lidar filtering.

The sensor information collected in the indoor environment was successively filtered using ConditionalRemoval, VoxelGrid, and RadiusOutlierRemoval, and the changes in the number of point clouds are shown in Figure 9. From the original, more than 60,000 point clouds were filtered to yield more than 2000 point clouds, thereby improving the speed of system mapping.

**Figure 10:**
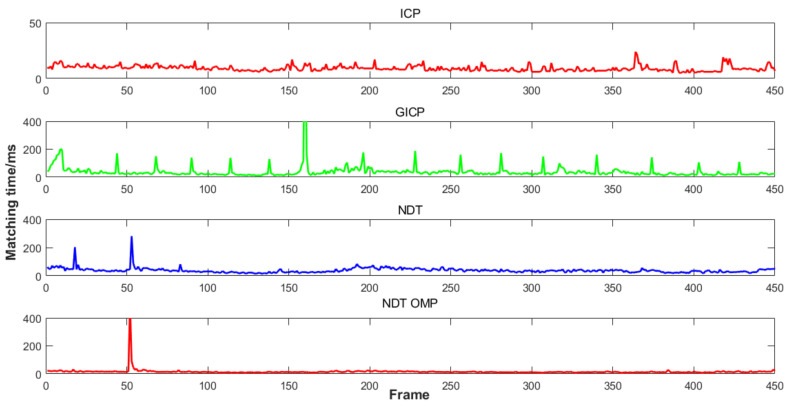
Time consumption of four inter-frame matching algorithm.

The comparison results of the running time of the four inter-frame matching algorithms in the indoor environment are shown in Figure 10. The ICP algorithm and NDT_OMP algorithm performed better according to the evaluation criterion of time consumption.

**Figure 11:**
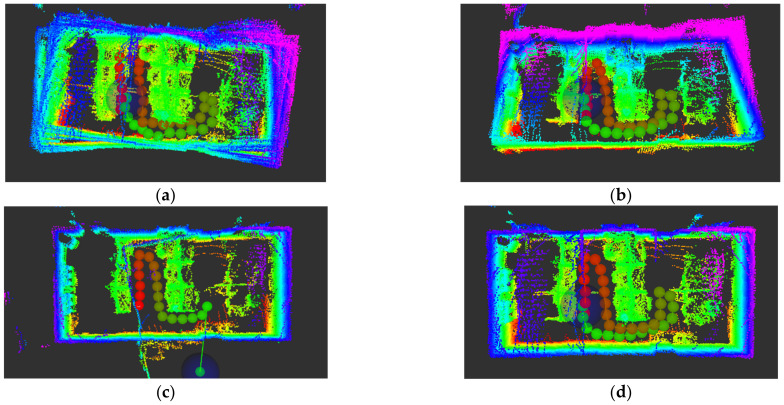
Drawing effect of four matching algorithms. (**a**) ICP-mapping, (**b**) GICP-mapping, (**c**) NDT-mapping, (**d**) NDT_OMP-mapping.

Figure 11 shows the construction effects of four types of inter-frame matching in an indoor environment.

**Figure 12:**
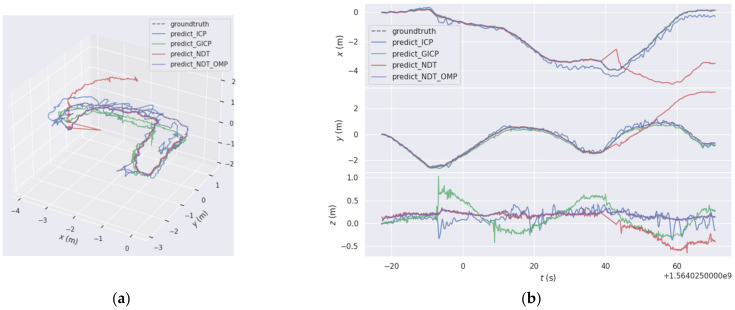
(**a**) Three-dimensional trajectory diagram and (**b**) *XYZ* coordinate data.

**Figure 13:**
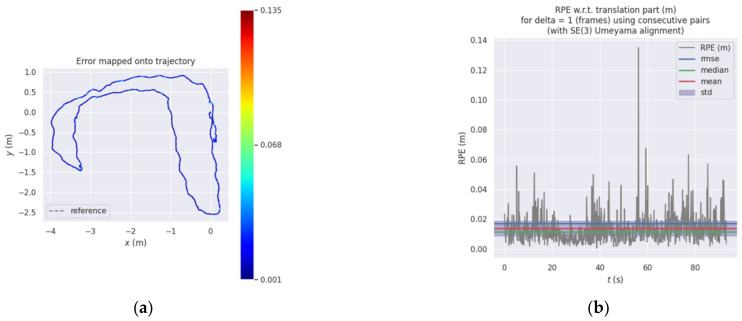
(**a**) Three-dimensional trajectory relative error and (**b**) specific value of relative error.

In the indoor environment, the 3D space track effect and the *XYZ* direction track coordinate values are shown in Figure 12. The figure shows the trajectories of the four matching algorithms in 3D space and the views in the *XYZ* direction. It can be seen from the figure that the ICP algorithm had a serious drift phenomenon. The predicted trajectories of the NDT_OMP algorithm were closer to the reference trajectories, and the mapping effect of the NDT_OMP algorithm was the best. Figure 13 shows the relative error of the predicted trajectory and the reference trajectory using the NDT_OMP algorithm in an indoor environment. It can be observed that the ICP algorithm had a huge elevation error. The relative error of the NDT_OMP algorithm with respect to the reference trajectory was within the acceptable range, and there were no sections with a large error. The results show that the NDT_OMP algorithm could meet the real-time accuracy of map construction in an indoor environment.

### 4.2. Path Planning Experiment

**Figure 14:**
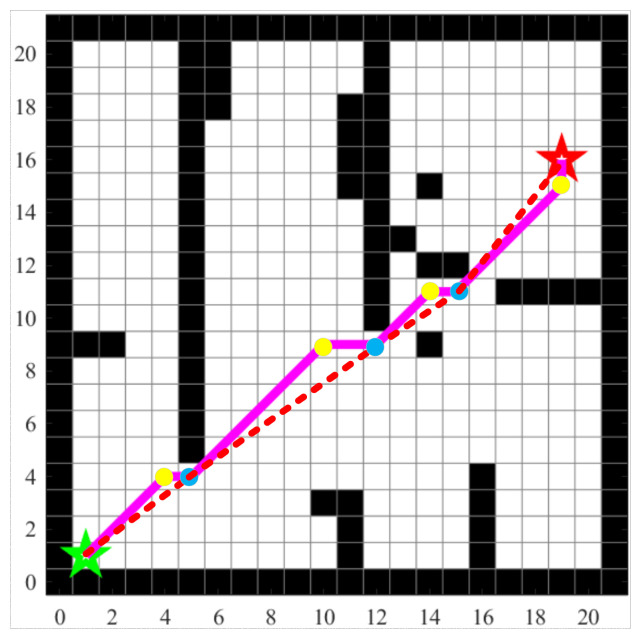
JPS algorithm path planning result. Black represents obstacles. The green five-pointed star represents the starting point, the red five-pointed star represents the target point, and the pink solid line represents the path planning result of the JPS algorithm. The red dashed line represents the connection between nodes. Yellow dots represent path planning nodes.

Many scholars have improved the A* algorithm, e.g., the JPS (jump point search) algorithm, which improves the search speed of path planning by reducing the operation of OpenList tables. However, through experiments, we found that there were redundant points in the final path planned by the JPS algorithm. In a large map, the path planned by the JPS algorithm would increase the work of the mobile robot. Figure 14 shows a result of the JPS algorithm. Looking at Figure 14, it can be seen that the red dashed line had a shorter path length than the pink solid line; therefore, the yellow points were redundant nodes, which could be removed in the path set, thereby reducing the path length and the power consumption of the mobile robot in the process of moving. The improved algorithm proposed in this paper can remove such redundant points and simplify the nodes of the path set. In addition, the A* algorithm is extensible and can be applied to grid map, Navmesh, and Waypoint nodes, while the JPS algorithm is only applicable to grid map nodes.

**Figure 15:**
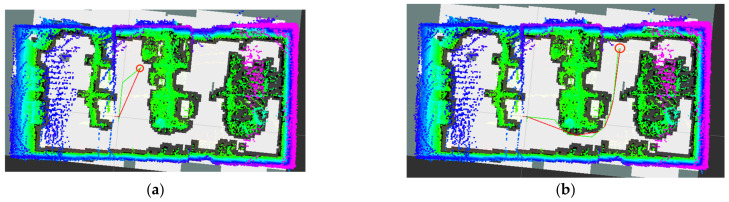
Example path planning diagram. (**a**) The first type of path planning, (**b**) The second type of path planning. The coordinate system position represents the starting position of the mobile robot, the red circle represents the target position, the green path is used to represent the result of the original A* algorithm, and the red path represents the result of the improved A* algorithm.

The improved A* algorithm proposed in this paper was applied to the constructed laboratory graph. After the host computer program created the map, the mobile robot waited for the host computer to send the location information of the target point, and then carried out autonomous path planning according to its current position and the location of the target point. Figure 15 shows the results. It can be observed that the inflection point of the red path was significantly smaller than that of the green path, effectively reducing the number of nodes in the path set. Compared with the traditional A* algorithm, the improved algorithm proposed in this paper achieved better results in terms of search speed and path smoothness. It can be seen that the path planned by the improved A* algorithm was more in line with the operability of the actual mobile robot.

Table 2 shows the search time of mobile robot path planning and the number of search extension nodes, where A* represents the original A* algorithm, and A* + turn_w represents the algorithm improved by adding the steering cost function, A* + turn_w + W represents the algorithm improved by adding the steering cost function and weight coefficient, time indicates the time consumed by the algorithm in ms, number indicates the number of extension nodes searched by the algorithm, Tir represents the time performance improvement rate of the algorithm compared with the original A* algorithm as a percentage, and Enr represents the improvement rate of the algorithm in search extension node performance compared with the original A* algorithm as a percentage.

It can be seen from Table 2 that the performance of the improved A* algorithm was improved in terms of search time and the number of extended nodes. The safety protection mechanism, path smoothing strategy, steering work function, and dynamic weight coefficient were introduced to optimize the traditional A* algorithm, thereby improving the planned path performance, bringing it more in line with the actual scene of mobile robot operation.

## 5. Discussion

In this paper, aiming at real-time and accurate mobile robot path planning, the indoor environment map was constructed using the Hdl_graph_slam mapping algorithm, and the path planning of a mobile robot was realized using an improved A* algorithm. The average path search time was decreased by 13%, and the average number of search extension nodes was decreased by 11%. According to results of this study, the accuracy, reliability, and operability of the proposed method can meet daily needs.

## Figures and Tables

**Table 1:** Coordinate transformation and turn angle mapping table.

*X*-axis Change	*Y*-axis Change	Steering Angleθ (°)
1	0	0
1	1	45
0	1	90
−1	1	135
−1	0	180
−1	−1	225
0	−1	275
1	−1	360

**Table 2:** Performance comparison of three different path planning algorithms.

Start and Finish	The Algorithm Name	Time (ms)	Number	Tir (%)	Enr (%)
Start (109, 89) Finish (71, 88)	A*	1.817	12,988	-	-
	A* + turn_w	1.699	12,408	8.145	4.465
	A* + turn_w + W	1.625	11,338	10.566	12.704
Start (109, 89) Finish (108, 33)	A*	4.090	22,322	-	-
	A* + turn_w	3.687	21,336	9.853	4.417
	A* + turn_w + W	3.362	20,072	17.799	10.079
Start (109, 89) Finish (125, 126)	A*	2.385	16,007	-	-
	A* + turn_w	2.168	15,728	9.098	1.742
	A* + turn_w + W	1.926	14,310	11.162	10.601

## Data Availability

The data are not publicly available due to the project requirements.
